# A Human Pan-Cancer System Analysis of Procollagen-Lysine, 2-Oxoglutarate 5-Dioxygenase 3 (PLOD3)

**DOI:** 10.3390/ijms22189903

**Published:** 2021-09-14

**Authors:** Siming Gong, Yingjuan Duan, Changwu Wu, Georg Osterhoff, Nikolas Schopow, Sonja Kallendrusch

**Affiliations:** 1 Institute of Anatomy, University of Leipzig, Liebigstraße 13, 04103 Leipzig, Germany; siminggong23@gmail.com (S.G.); nikolas.schopow@medizin.uni-leipzig.de (N.S.); sonja.kallendrusch@medizin.uni-leipzig.de (S.K.); 2 Faculty of Chemistry and Mineralogy, University of Leipzig, 04103 Leipzig, Germany; yingjuan0103@gmail.com; 3 Sarcoma Center, Department of Orthopedics, Trauma and Plastic Surgery, University Hospital Leipzig, 04103 Leipzig, Germany; georg.osterhoff@medizin.uni-leipzig.de

**Keywords:** PLOD3, pan-cancer, prognosis, tumor, immune infiltration, enrichment analysis, immunotherapy, big data

## Abstract

The overexpression of the enzymes involved in the degradation of procollagen lysine is correlated with various tumor entities. Procollagen-lysine, 2-oxoglutarate 5-dioxygenase 3 (PLOD3) expression was found to be correlated to the progression and migration of cancer cells in gastric, lung and prostate cancer. Here, we analyzed the gene expression, protein expression, and the clinical parameters of survival across 33 cancers based on the Clinical Proteomic Tumor Analysis Consortium (CPTAC), function annotation of the mammalian genome 5 (FANTOM5), Gene Expression Omnibus (GEO), Genotype-Tissue Expression (GTEx), Human Protein Atlas (HPA) and The Cancer Genome Atlas (TCGA) databases. Genetic alteration, immune infiltration and relevant cellular pathways were analyzed in detail. PLOD3 expression negatively correlated with survival periods and the infiltration level of CD8^+^ T cells, but positively correlated to the infiltration of cancer associated fibroblasts in diverse cancers. Immunohistochemistry in colon carcinomas, glioblastomas, and soft tissue sarcomas further confirm PLOD 3 expression in human cancer tissue. Moreover, amplification and mutation accounted for the largest proportion in esophageal adenocarcinoma and uterine corpus endometrial carcinoma, respectively; the copy number alteration of PLOD3 appeared in all cancers from TCGA; and molecular mechanisms further proved the effect of PLOD3 on tumorigenesis. In particular, PLOD3 expression appears to have a tumor immunological effect, and is related to multiple immune cells. Furthermore, it is also associated with tumor mutation burden and microsatellite instability in various tumors. PLOD3 acts as an inducer of various cancers, and it could be a potential biomarker for prognosis and targeted treatment.

## 1. Introduction

The expression of procollagen-lysine, 2-oxoglutarate 5-dioxygenase 3 (PLOD3) is essential for the biosynthesis of collagen and gene mutations thereof, and are related to disorders of the connective tissue [1,2,3]. Clinical related mutations with reduced expressions of the PLOD family are associated with the Ehlers-Danlos and Bruck syndromes [4,5,6]. PLOD3 alterations during tissue repair might, thus, lead to epithelial–mesenchymal transition (EMT). This process describes the transformation of stable epithelial cells undertaking phenotypic transformation. Consequently, cell to cell adherence as well as cell polarity are altered, gaining the ability to migrate and invade novel territories. EMT is a key process in wound healing, tissue repair and, also, in the development of a variety of cancers [7,8,9].

Among others, fibroblasts, chondroblasts and osteoblasts produce procollagen. Many post-translational modifications are necessary to generate collagens, which build a triple-helical molecule. PLOD3 belongs to a family of enzymes that is able to catalyze the lysine hydroxylation of procollagen using Fe^2+^. However, an upregulation of PLOD3 was recently found in fibrosis and solid tumors with poor prognosis [3,10,11].

Current literature suggests a strong relation between collagen modulation and cancer [3,10]. In this pan-cancer system analysis, PLOD3 expression is being investigated in a broad variety of human solid tumor data, available within the Clinical Proteomic Tumor Analysis Consortium (CPTAC), function annotation of the mammalian genome 5 (FANTOM5), Gene Expression Omnibus (GEO), Genotype-Tissue Expression (GTEx), Human Protein Atlas (HPA) and The Cancer Genome Atlas (TCGA) databases.

The study analyzes gene alteration information, protein expression, prognostic ability and gene immune analysis of the PLOD3 of more than 10,000 samples from several comprehensive databases. Furthermore, we performed an enrichment analysis of related genes to investigate the potential role and molecular mechanism of PLOD3.

This study aims to understand the role and potential mechanism of PLOD3 in the process of oncogenesis of 33 tumors in humans.

## 2. Results

### 2.1. Experiment Setup and Genetic Alteration Analysis Data

**Figure 1:**
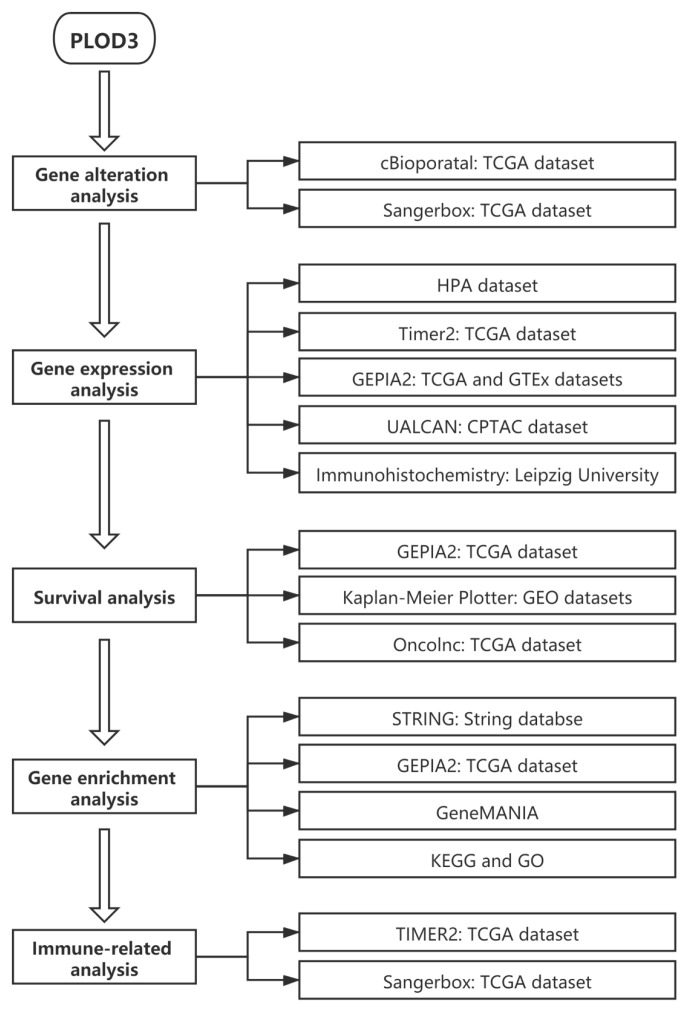
Setup of the integrative and comprehensive pan-cancer analysis of PLOD3.

Figure 1 shows the setup of this study for the tumors, including: adrenocortical carcinoma (ACC), breast invasive carcinoma (BRCA), bladder urothelial carcinoma (BLCA), colon adenocarcinoma (COAD), cholangiocarcinoma (CHOL), cervical squamous cell carcinoma and endocervical adenocarcinoma (CESC), lymphoid neoplasm diffuse large B-cell lymphoma (DLBC), esophageal carcinoma (ESCA), glioblastoma multiforme (GBM), head and neck squamous cell carcinoma (HNSC), kidney chromophobe (KICH), kidney renal clear cell carcinoma (KIRC), kidney renal papillary cell carcinoma (KIRP), liver hepatocellular carcinoma (LIHC), lower grade glioma (LGG), acute myeloid leukemia (LAML), lung adenocarcinoma (LUAD), lung squamous cell carcinoma (LUSC), mesothelioma (MESO), ovarian serous cystadenocarcinoma (OV), prostate adenocarcinoma (PRAD), pheochromocytoma and paraganglioma (PCPG), pancreatic adenocarcinoma (PAAD), rectum adenocarcinoma (READ), sarcoma (SARC), stomach adenocarcinoma (STAD), skin cutaneous melanoma (SKCM), thymoma (THYM), thyroid carcinoma (THCA), testicular germ cell tumors (TGCT), uterine carcinosarcoma (UCS), uterine corpus endometrial carcinoma (UCEC) and uveal melanoma (UVM). 

**Figure 2:**
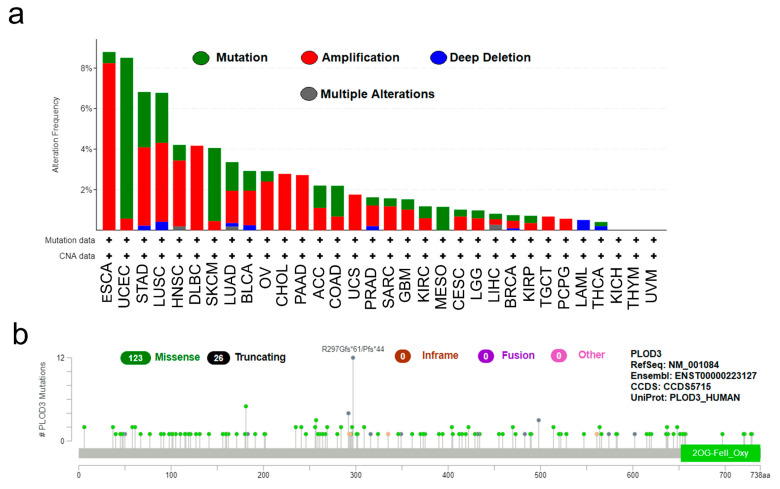
PLOD3 mutation feature in different tumors of TCGA. The alteration frequency and the type is shown in (**a**); The mutation types, sites and frequency of PLOD3 genetic alteration are displayed in (**b**).

Figure 2a illustrates the mutation, amplification, deep deletion and multiple alterations frequency of the PLOD3 gene and the copy number alteration (CNA) across different cancers. The CNA of PLOD3 occurred in all of these cancers except KICH, THYM and UVM. In ESCA, amplification accounted for the largest proportion (8.2%), while in UCEC and SKCM, the largest part was mutation (7.9% and 3.6%). 

The diagram of mutation sites, additionally, explains the different types and the case number. Missense was the most common type of mutation with the number of 123, followed by truncating mutation with the number 26 (Figure 2b).

### 2.2. Gene Expression Analysis Data 

In the beginning, the expression level of PLOD3 in healthy tissue were analyzed based on GTEx, HPA, and FANTOM5. Supplementary Figure S1a illustrates that PLOD3 expressions are highest in human liver, vaginal and midbrain tissue. However, PLOD3 is expressed in most origins but showed low RNA specificity between different tissues. This low RNA specificity was also detected in the variable blood cells, based on a combination of the three datasets (Supplementary Figure S1b).

**Figure 3:**
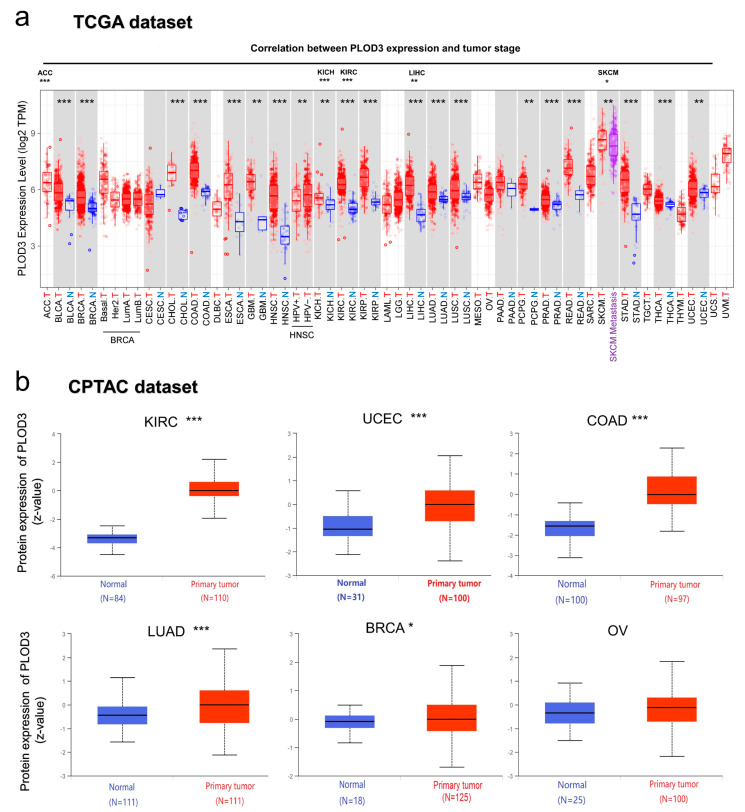
PLOD3 expression in different cancers. (**a**) The PLOD3 expression level in different cancers or specific cancer subtypes. N—normal and T—tumor tissue; (**b**) The PLOD3 total protein expression between tumor tissue and corresponding normal tissue according to the CPTAC dataset. * *p* < 0.05; ** *p* < 0.01; *** *p* < 0.001.

The expression of the PLOD3 gene was analyzed in the context of the TCGA database. Figure 3a shows that PLOD3 expression is higher in tumor tissues than in adjacent normal tissues across different cancers, such as BLCA, BRCA and CHOL (all *p* < 0.05). However, some tumor entities (complete ACC, DLBC, LGG, limited PCPG) are missing sufficient information on healthy origin tissues in the respective dataset of TCGA. 

Compared to normal tissue samples from the GTEx dataset serving as controls, PLOD3 expression is higher in some tumors, such as ACC, DLBC and LGG (Supplementary Figure S2a), but no significant difference was seen for LAML, OV, SARC and UCS (Supplementary Figure S2b). 

In the CPTAC dataset, PLOD3 total protein expresses higher in primary cancers than in normal tissues for COAD, KIRC, UCEC, BRCA and LUAD, while the expression in ovarian cancer shows no increased protein expression (Figure 3). 

**Figure 4:**
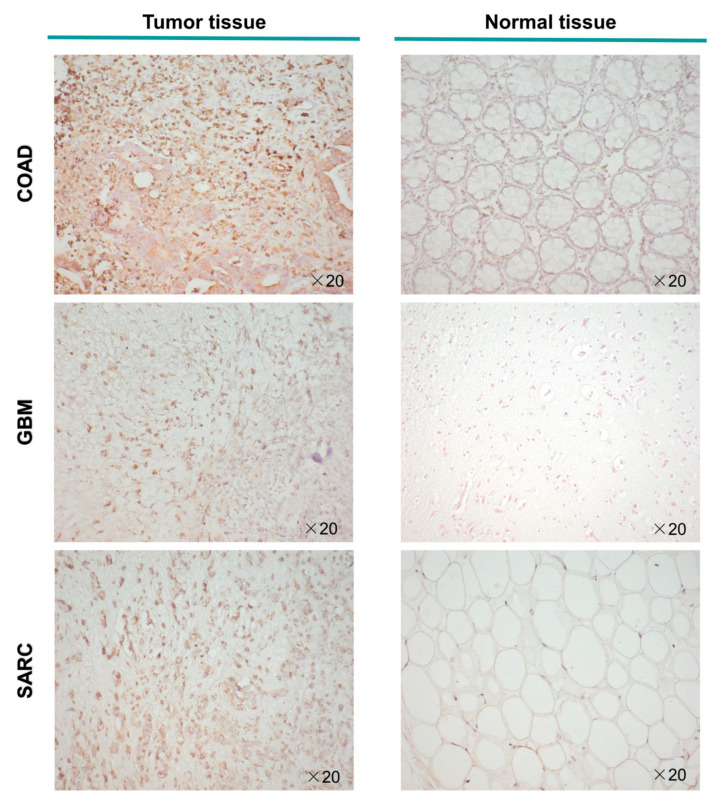
Immunohistochemical staining using patient derived tissue: PLOD3 expression in human COAD, GBM and SARC compared with corelating normal tissue (colon, brain, and adipose tissue).

Representative PLOD3 immunohistochemistry staining is shown in Figure 4. It illustrates that the expression of PLOD3 from patient derived COAD, GBM, and SARC tissue was higher compared to the expression in corresponding healthy human tissue. In particular, the staining shows PLOD3 expression in the stromal cell populations of the TME, not of the tumor cells themselves.

Additionally, the expression of PLOD3 was related to pathological stages in ACC, KICH, KIRC, LIHC and SKCM, but not significantly related to the stages of other cancers (Supplementary Figure S3). 

### 2.3. Survival Analysis Data

**Figure 5:**
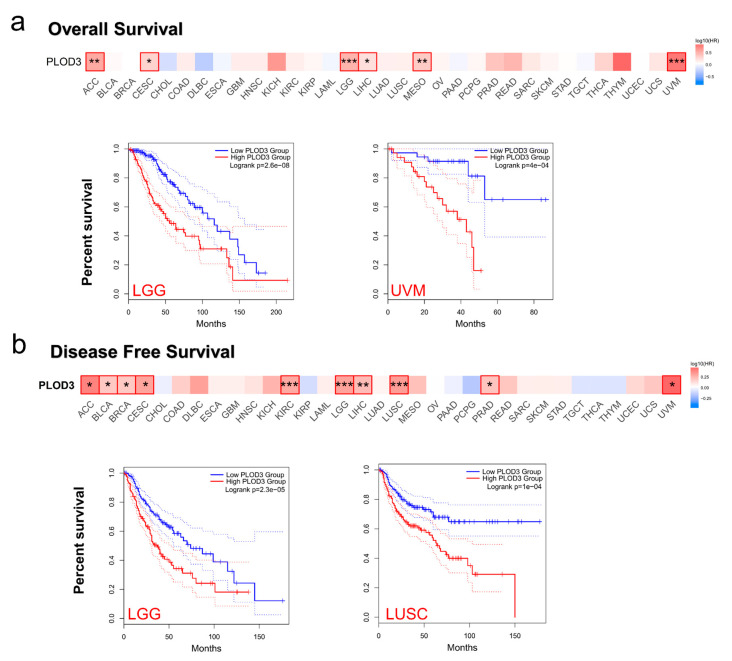
Relation between *PLOD3* expression and survival prognosis of different cancers in TCGA. GEPIA2 tool was used to obtain the OS (**a**) and DFS (**b**) analyses of different tumors in TCGA. The survival maps and Kaplan–Meier curves are shown. Dotted lines: 95% confidence interval. * *p* < 0.05, ** *p* < 0.01, *** *p* < 0.001.

We separated the TCGA database into two groups. High and low PLOD3 expression and tumor prognosis was compared. Figure 5 shows overall survival (OS) and disease-free survival (DFS) for different kinds of tumors. 

The survival map shows the relation between PLOD3 expression and OS. A high expression of PLOD3 was associated with poor OS in some cancers, namely, in ACC, CESC, LGG, LIHC, MESO and UVM (all *p* < 0.05, Figure 5a). Figure 5b demonstrates the negative correlation between PLOD3 expression and DFS in ACC, BLCA, BRCA, CESC, KIRC, LGG, LIHC, LUSC, PRAD and UVM (all *p* < 0.05). In the Kaplan–Meier curves, the correlation between the high and low expression groups was exemplified for OS of LGG and UVM and for DFS of LGG and LUSC (Figure 5).

Supplementary Figures S4 and S5 also illustrate that the high expression of PLOD3 was associated with poor prognosis in cancers such as KIRC, LUAD, OV and READ.

PLOD3 expression is negatively correlated with different prognosis periods: e.g., breast cancer: overall, relapse free and distant metastasis free survival; or lung cancer: first progression, overall and postprogression survival (all *p* < 0.05, Supplementary Figure S6 also shows gastric, liver and ovarian cancer).

### 2.4. Analysis of Related Genes Network 

**Figure 6:**
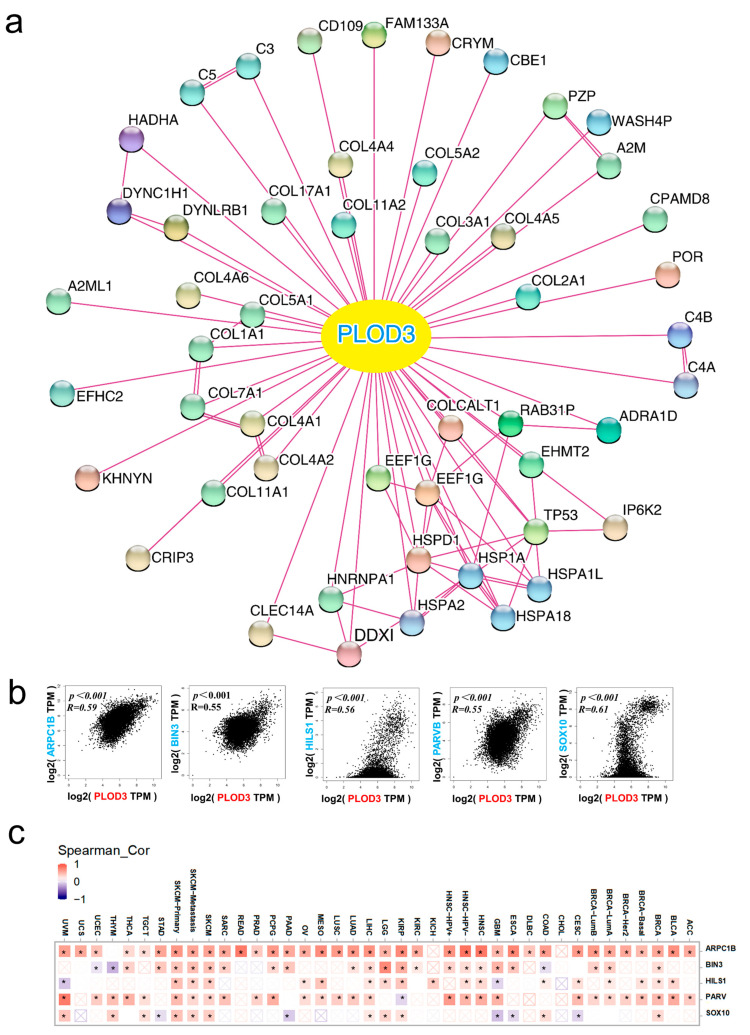
PLOD3 related gene enrichment analysis. (**a**) STRING tool was used to obtain the available, experimentally determined PLOD3-related proteins; (**b**) The GEPIA2 approach was employed to obtain the top 100 PLOD3-correlated genes in TCGA database and the relation between PLOD3 and selected targeting gene expressions were analyzed. The top five genes are shown; (**c**) The corresponding heatmap data in all investigated cancer types are displayed. * *p* < 0.05.

For a deeper exploration of the potential mechanisms behind PLOD3 in oncogenesis, we observed the top 50 PLOD3-interacting proteins and carried out a protein–protein interaction network (Figure 6a) and obtained the positive relation of PLOD3 with top 100 PLOD3 correlated genes. The top 5 were: SRY-Box Transcription Factor 10 (SOX10) (R = 0.61), Actin Related Protein 2/3 Complex Subunit 1B (ARPC1B) (R = 0.59), Spermatid-specific linker histone H1-like protein (HILS1) (R = 0.56), Parvin Beta (PARVB) (R = 0.55) and Bridging Integrator 3 (BIN3) (R = 0.55) (Figure 6b, all *p* < 0.05).

Figure 6c presents the positive relation between PLOD3 expression level, with ARPC1B, HILS1, PARVB and BIN3 in most cancers from TCGA database, and a positive correlation with SOX10 in some of the cancers.

### 2.5. KEGG Pathway and GO Enrichment Analysis Data

**Figure 7:**
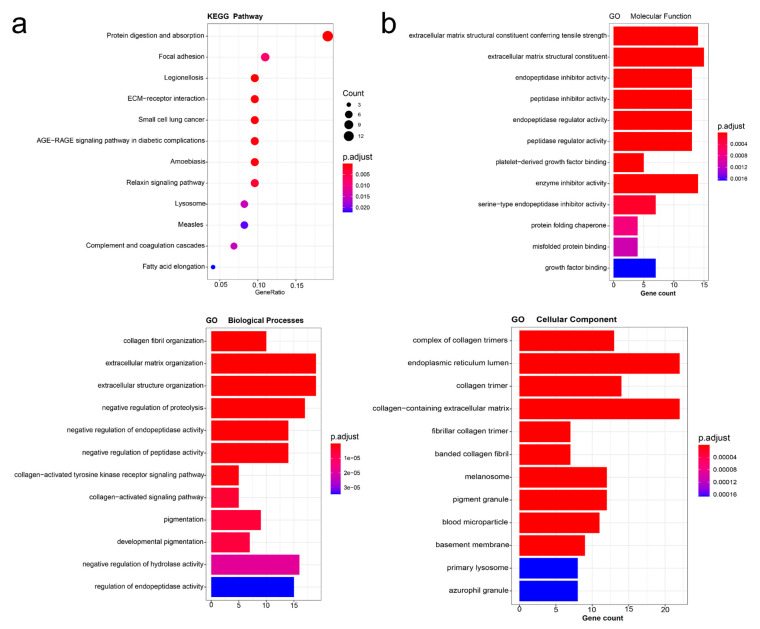
The enrichment analysis of PLOD3 and most frequent neighboring genes. (**a**) KEGG pathway analysis was obtained according to PLOD3-binding and interacted genes; (**b**) Bar plot of GO enrichment in cellular component terms, biological process terms, and molecular function terms.

Kyoto Encyclopedia of Genes and Genomes (KEGG) pathway analysis shows that the expression of PLOD3 is associated with many factors, and the PLOD3 gene could be involved in oncogenesis and cancers metastasis by “protein digestion and absorption”, “focal adhesion” and “small cell lung cancer” (Figure 7a). 

Gene Ontology (GO) enrichment analysis (Figure 7b) indicated that PLOD3 and related genes were mainly associated with cellular components, such as endoplasmic reticulum lumen, biological processes such as extracellular matrix organization, collagen-activated tyrosine kinase receptor signaling pathway and regulation of endopeptidase activity, and molecular function, e.g., protein folding chaperone and misfolded protein binding.

### 2.6. Immune Infiltration Analysis Data

**Figure 8:**
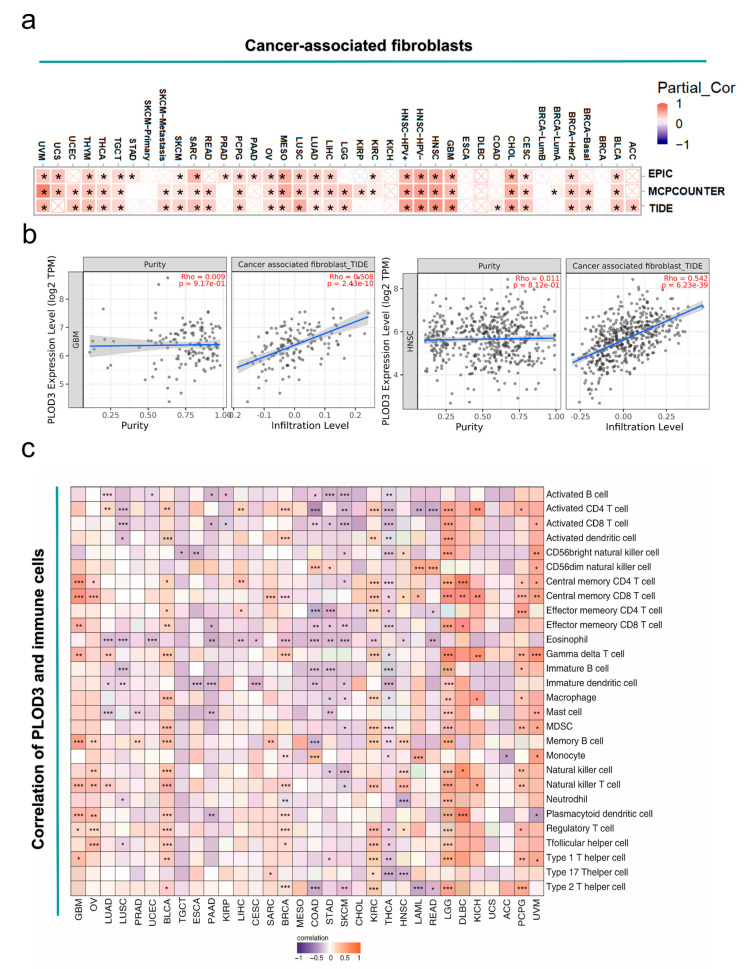
The analysis of relation between PLOD3 expression, immune infiltration of cancer associated fibroblasts and the immune cells. (**a**) Three kinds of algorithms were used to explore the possible correlation between the expression level of the PLOD3 gene and the infiltration level of cancer associated fibroblasts across serious types of cancer in TCGA; (**b**) Scatter plots of cancer associated fibroblasts immune infiltration in different tumors generated based on a certain algorithm; (**c**) Association between PLOD3 expression and different immune cells in different tumors. * *p* < 0.05, ** *p* < 0.01, *** *p* < 0.001.

The heatmap shows correlations between PLOD3 expression and cancer associated fibroblasts (CAF) across diverse cancers of TCGA database. These analyses, based on the algorithms EPIC, MCPCOUNTER and TIDE, and the correlations with the same trend in all three were considered to be plausible. The expression of PLOD3 is positively related to CAF for most of the mentioned cancers, such as BLCA, SARC and CESC (Figure 8a). The scatter plot also illustrates the positive relation of PLOD3 expression and CAF in the aforementioned cancers. Based on the algorithms, cancers with the strongest correlation were shown (Figure 8b). 

The Sangerbox tool was used to obtain the immune maps that could show the relation between the expression of PLOD3 and immune cell infiltration in a tumor microenvironment (TME). In total, 28 kinds of immune cells were included in the immune cells map, such as activated B cell, activated CD4^+^ T cell and activated CD8^+^ T cell. The immune map showed that the expression of PLOD3 in BLCA, KIRC and LGG was positively correlated with most of the immune cells, while THCA was negatively corelated with most of the immune cells. The results are shown in Figure 8c.

**Figure 9:**
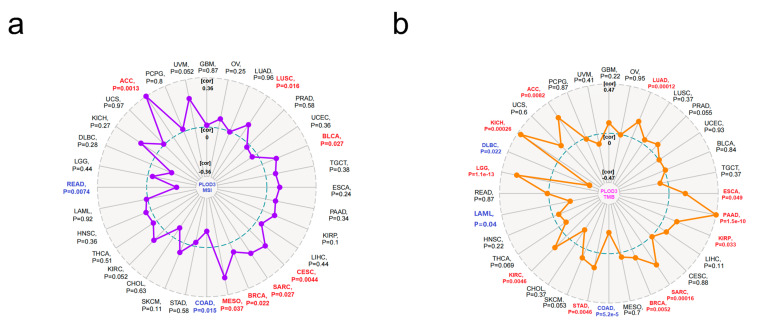
Correlation between PLOD3 expression and microsatellite instability (MSI) or tumor mutational burden (TMB). Based on TCGA database, MSI and TMB were obtained. The *p*-value is supplied. (**a**) The potential correlation between PLOD3 expression and MSI. The partial correlation (Cor) values of +0.36 and −0.36 are marked; (**b**) The potential correlation between PLOD3 expression and TMB. The partial correlation (Cor) values of +0.47 and −0.47 are marked.

In addition, we also investigated the relation of PLOD3 expression to the tumor mutational burden (TMB) and microsatellite instability (MSI), respectively, in different cancers from the TCGA database. The PLOD3 expression is positively related to TMB for KICH, LGG, KIRC, STAD, BRCA, SARC, KIRP, PAAD, ESCA, LUAD and ACC, whereas the expression of PLOD3 is negatively related to TMB for DLBC, LAML and COAD (all *p* < 0.05). The high expression of PLOD3 is associated with high MSI in ACC, MESO, BRCA, SARC, CESC, BLCA and LUSC, but correlates with low MSI in READ and COAD (all *p* < 0.05) (Figure 9).

Supplementary Figure S7a demonstrates the relation between PLOD3 expression level and CD8^+^ T cell infiltration level via ten algorithms (e.g., TIMER, EPIC and MCPCOUNTER). PLOD3 expression level was negatively correlated with CD8^+^ T cell infiltrate level in PAAD and SKCM on the basis of most of the algorithms (8/10 and 9/10). The scatter plots generated according to the strongest correlative algorithms are shown in Supplementary Figure S7b.

## 3. Discussion

The PLOD3 gene is critical for collagen synthesis, thus, mutations are related with disorders of the connective tissue and the development of several tumor entities [1,2,5]. In this study, more than 10,000 samples from the CPTAC, FANTOM5, GEO, GTEx, HPA and TCGA databases were analyzed. Tests were performed for gene alteration, gene expression, survival analysis and immune infiltration. Furthermore, a gene enrichment analysis was carried out, to reveal the potential significance of PLOD3 gene regulation in cancer.

In most of the analyzed cancers, PLOD3 gene expression was significantly higher in tissues of cancer compared to its expression in healthy tissues (Figure 3). The overexpression of the PLOD3 gene was further linked to poor clinical prognoses (e.g., ACC, LGG, LIHC, Figure 5). This observation, indicating the cancer-promoting abilities of PLOD3, are in line with previously published studies, suggesting that the expression of PLOD3 could lead to oncogenesis and cancer metastasis in a variety of tumors [3,7,10,11]. For example, PLOD3 can promote lung cancer metastasis by regulating STAT3, and PLOD3 silencing can inhibit the proliferation of glioma cells via P21 pathway [12,13]. Analyzing the genetic alteration of PLOD3 in our study, it is observable that the mutation and CNA contribute, to a great extent, to diverse cancers. Previous studies have shown that the mutation of PLOD3 can lead to disorders of connective tissue disease and other diseases [1,2,14,15]. In addition, it is generally known that genetic alteration plays a key role in the development of tumors [16,17]. In this study, PLOD3 amplification is underrepresented in cancers such as UCEC and SKCM, while mutations occur most often (7.94% and 3.9%). Therefore, one could assume that the mutations of the PLOD3 gene are part of the oncogenesis.

Two possible mechanisms that could account for the observed effects are discussed in the literature. One mechanism relates to the extracellular matrix, a component of the cellular microenvironment that is composed of structural proteins, glycoproteins and proteoglycans. As collagen is one of the main components of the extracellular matrix, it takes part in regulating cancer cell invasion, metastasis and survival through biochemical and biophysical signals, resulting from collagen–cell interactions [15,18,19,20,21,22,23]. Lysyl Dydroxylase 3 (LH3), a protein highly involved in collagen biosynthesis, is encoded by PLOD3 [1,9,19]. Another possibility is somatcanCNA. Somatic changes influence a great part of the genome in cancer cells, and are involved in many kinds of genetic alteration, such as single base substitutions, translocations and infections, influencing oncogenes and tumor suppressor genes [23,24]. Therefore, understanding the mechanisms resulting in PLOD3 mutation, as well as its biological effects, might affect the diagnosis and therapy of cancer.

The String tool and GeneMANIA were used to reveal a comprehensive network of PLOD3 related genes with physical or functional interactions [25,26,27]. The 100 most highly correlated genes with PLOD3 were selected (Supplementary Table S1) and the expression of the top five was examined for 33 tumor entities (Figure 6b,c). Although no regulatory relationships between PLOD3 and the top five interacted genes have been reported in the literature, a potential regulatory network was still identified (Supplementary Figure S8). This may be conducive to further revealing the mechanism of PLOD3 in tumors in the future. In fact, previous studies indicate that ARPC1B is a component of the Arp2/3 complex, a polyprotein composite, which can mediate the formation of branch myocyanin networks in the cytoplasm, providing a driving force of cell motility [28,29]. PARVB is involved in the function of actin and plays a key role in cell adhesion, diffusion and migration, which suggests their potential role in EMT [30]. In contraction, PLOD3 is also closely related to EMT in our previous description, which further supports our conclusion. In addition, it is worth noting that HILS1 is also related to chromatin remodeling and transcriptional regulation [31], in view of its strong correlation with PLOD3 and the result of enrichment analysis, this may imply the potential role of PLOD3 in chromatin function. 

In the enrichment study, the KEGG pathway and the GO enrichment analysis especially deserve attention. The KEGG pathway shows that PLOD3 was related with protein digestion and absorption, focal adhesion, and small cell lung cancer, which could be a potential reason for the alteration of PLOD3 in disorders of the connective tissue, tumorigenesis, and cancer progression [1,2,3,4,7,10,11], this is worthy of further research. Previous research stated that proteins function as team players to form complex cellular machines and transmit signals within cells [32]. Protein–protein interactions affect cellular pathways in living organisms, thus, deciphering changes in the protein facilitates the understanding of tumorigenesis on a systematic level [32,33]. With respect to the cellular component of GO, the importance of the research on the endoplasmic reticulum lumen is supported by other studies [34,35]. High quality protein folding plays a vital role in cell survival and the normal physiology of the organism [35]. However, altered endoplasmic reticulum homeostasis causes the unfolded and misfolded proteins to accumulate in the endoplasmic reticulum lumen, which is known as endoplasmic reticulum stress and could facilitate a cell survival mode to restore homeostasis [36,37]. Chronic or severe endoplasmic reticulum stress, however, induces intracellular oxidative stress and impairs the mitochondrial function, which leads to cell apoptosis [37]. According to our enrichment study, PLOD3 related genes are involved in protein related processes, such as “endoplasmic reticulum lumen”, “protein folding chaperone” and “misfolded protein binding”, which could be one potential mechanism of tumor formation.

Our immunohistochemical analysis of various cancer tissues revealed glial cells and CAF as the main cellular population expressing PLOD3. CAFs are involved in regulating tumor-infiltrating immune cells, cancer initiation, progression, or metastasis [38,39,40,41]. These cells form one of the most necessary factors of the TME, playing a key role in the process of tumor infiltration, in promoting oncogenesis, and are associated with the immune evasion of cancer cells [42,43,44,45]. Research has shown that targeting CAF derived factors could be promising in patients with drug resistance to immune checkpoint inhibitors (ICIs) [44]. It is well known that CD8^+^ T cells play a crucial role in the antitumor effect of ICIs [46,47,48,49]. However, the CD8^+^ T cells are mostly in an “exhausted” condition due to prolonged inflammation in cancer [46,47,49]. This study shows that PLOD3 expression was negatively correlated to the infiltration degree of CD8^+^ T cells, but was positively related to CAFs in the following cancers, SKCM, LUSC and THCA (Figure 8). This result suggests that PLOD3 targeted drugs might be beneficial, in combination with immunotherapy, against these cancers. In COAD studies it was shown that CAF could be a factor affecting tumor initiation and enhanced drug resistance [50,51]. Some other studies show that CAFs play a role in the process of cancer metastasis and related PLOD3 to metastasis in LUAD [10,52,53]. 

Further, Wu et al. elucidated that high macrophage and neutrophile infiltration prominently reduced the prognosis of prostate cancer, indicating the possibility of the aforementioned two kinds of cells as drug targets for prostate cancer [41]. In this study, the PLOD3 gene was positively correlated with macrophage and neutrophile infiltration level in LGG, which may indicate that a target drug could be effective in LGG.

Immunotherapy is well implemented in the clinical practice of various cancers nowadays [54,55,56]. Previous studies have shown that the cytotoxic T lymphocyte associated protein-4 (CTLA-4) and programmed cell death protein-1 (PD-1) play an essential role in the development of cancer immunotherapy [57,58]. Recent research has shown the infiltration of immune cells in cancers and the possibility of these as prognostic biomarkers [40,59]. MSI is a hypermutable phenotype caused by the loss of DNA mismatch repair activity, and is used to predict prognosis of cancers [60,61]. TMB is also a biomarker to predict the response to immune target therapy [60,61,62,63,64]. Our study demonstrated that PLOD3 is positively correlated with the TMB in tumors such as LGG and KIRC. The immune map further shows that LGG and KIRC are positively related with most of the immune cells; together these results suggest, again, that targeting PLOD3 might be beneficial for immune therapy (Figure 9). Nevertheless, there are variabilities in certain cancer entities and a multigene analysis should address this limitation. The PLOD3 gene belongs to the PLOD gene family, and the PLOD gene family are related to each other (Supplementary Figure S6). However, using the PLOD gene family and PLOD3 protein expression might be a valuable marker for cancer prognosis.

Nevertheless, no therapy dependent effect could be detected in the current study. Baek et al. proved that PLOD3 knockdown could inhibit renal cell carcinoma malignance as well as lung tumor growth. In lung cancer cells, PLOD3 knockdown suppresses chemoresistance and radioresistance, which might promote the curative effect with chemotherapy and radiotherapy [65,66,67]. Considering the essential role of PLOD3, and the encoded enzyme, in the formation of the extracellular matrix derived from fibroblasts, chondroblasts and osteoblasts, we were not surprised that it could also be overexpressed in connective tissue malignancies (SARC), compared to corresponding normal tissue, according to the immunohistochemical analyses. Considering the findings of Jiang et al., that PLOD1 is a prognostic marker in osteosarcomas [68]. Further studies, especially of soft tissue sarcomas, are pending. In future research, we suggest exploring whether PLOD3 expression relates to the sensitivity of therapeutic interventions, such as radio-, immuno- and chemotherapy across various types of cancers. 

Taken together, our findings elucidate that PLOD3 could act as an inducer of various cancers, PLOD3 expression possessed a negative correlation with the survival odds and the infiltration level of CAF and a negative relation to the CD8^+^ T cells infiltration, to a certain extent. Hence, we may identify PLOD3 as a prognostic biomarker and a potential therapeutic target for some cancers, in an attempt to improve survival probability.

## 4. Materials and Methods

### 4.1. Genetic Alteration Analysis

“Cancer Types Summary” module of the cBioPortal was used to obtain the PLOD3 gene data and we entered the PLOD3 gene in the “TCGA Pan Cancer Atlas Studies” module to obtain the tumor entities summary, alteration frequency and CNA data. 

The “mutation” module was chosen to gain the diagram of PLOD3 alteration sites that included the alternation types and number. 

### 4.2. Gene expression Analysis

After entry into the tumor immune estimation resource, version 2 (TIMER2) (http://timer.cistrome.org/, accessed on 20 May 2021), PLOD3 gene was studied to gain discrepancies in its expression, between cancer and normal tissue, for various cancers or particular subtypes of cancers, according to the data from TCGA database. Then, the gene expression profiling interactive analysis, version 2 (GEPIA2) (http://gepia2.cancer-pku.cn/#analysis, accessed on 20 May 2021), was employed to analyze the expression of PLOD3 in cancers where only tumors or limited normal cases existed in the last step; hereby, the box plot was obtained for those cancers and their adjacent normal tissues by matched TCGA normal and GTEx data and default of log2FC (fold change) cutoff, *p*-value cutoff. Then, the violin plot was obtained to explore the PLOD3 expression in dissimilar pathological stages for types of cancers from TCGA database. In addition, UALCAN (http://ualcan.path.uab.edu/analysis-prot.html, accessed on 20 May 2021) was used to assess the PLOD3 total protein for cancers such as BRCA, UCEC and LUAD, on the basis of clinical proteomic tumor analysis consortium (CPTAC) dataset.

Moreover, the HPA database (https://www.proteinatlas.org/humanproteome/pathology, accessed on 20 May 2021) was used to obtain the expression data of the PLOD3 gene in various cells and tissues under physiological conditions.

Patient derived cancer tissues were obtained from University Hospital Leipzig. Three different cancers (COAD, GBM and SARC) were chosen in this study. For each entity, tissue samples from three patients were stained by 3,3′-diaminobenzidine tablets (DAB, Sigma Aldrich, St. Louis, MO, USA). Immunohistochemical staining was carried out as described previously [69]. The following antibody were used: PLOD3 antibody (11027-1-AP, Lot 00059310, 1:100 dilution, ProteinTech Group, Chicago, IL, USA).

### 4.3. Survival Prognosis Analysis

Survival maps of PLOD3 were obtained for diverse cancers from the TCGA database through “50% cutoff-high and 50% cutoff-low” on the GEPIA2 to separate into high and low expression groups. Then, “survival analysis” was acquired to evaluate the effect of PLOD3 expression on OS and DFS across different tumors that had statistical significance in last step, herein, the log-rank was applied.

Kaplan–Meier plotter (http://kmplot.com/analysis/, accessed on 25 May 2021) was used to analyze the OS, distant metastasis free, relapse free, postprogression, disease specific, and progress free survival, and first progression of lung, ovarian, gastric, and liver cancers based on “auto select best cutoff”.

### 4.4. Construction of Related Genes Network

The STRING (https://string-db.org/, accessed on 25 May 2021) was employed to determine the PLOD3 interacted proteins with input PLOD3 and the application of following parameters: “evidence” for “meaning of network edges”, “experiments” for “active interaction sources” and “disable structure previews inside network bubbles” for “network display options”. GeneMania (https://genemania.org/, accessed on 15 August 2021) was also used to further construct the interactive network of the top 5 PLOD3 interacted proteins. Then, the top 100 PLOD3 related genes were acquired on the “similar genes detection” module of GEPIA2. Picked genes were, respectively, used to conduct a dot plot for the correlation analysis through the application of Pearson correlation and log2 TPM and the mark of *p*-value and the correlation coefficient (R). In addition, the chosen genes were entered into the “Gene_Corr” module of TIMER2 to perform a heatmap for the correlation analysis with PLOD3 for all TCGA database cancers based on the purity adjustment and indication of the partial correlation (cor) and *p*-value. In addition, the GeneMANIA tool (https://genemania.org/, accessed on 15 August 2021) was used to obtain the network of PLOD3 and related genes.

### 4.5. KEGG Pathway and GO Enrichment Analysis 

The data of PLOD3 related genes were combined to KEGG pathway and GO analysis with following method: R package “clusterProfiler” in R software (Version 4.0.3, R Foundation for Statistical Computing, Vienna, Austria) was used to perform KEGG pathway and GO enrichment analysis including biological process, cellular component, and molecular function.

### 4.6. Immune Infiltration Analysis

“PLOD3” and “cancer associated fibroblast” were entered on the “immune-gene” of TIMER2 to obtain a heatmap about the correlation analysis between PLOD3 gene expression and CAF across all cancers in TCGA database on the basis of purity adjustment and three algorithms, namely, EPIC, MCP-COUNTER and TIDE, in this process the negative or positive correlation were shown with the *p*-values and correlation values. Then, the scatter plots were acquired to assess the specific correlation condition for the cancers where PLOD3 possessed positive correlation with the cancer associated fibroblast via all three algorithms based on the last step.

The relation between the PLOD3 gene expression and the CD8^+^ T cells infiltration was analyzed via the “Immune-Gene” of TIMER2 based on some algorithms (e.g., TIMER, EPIC, MCPCOUNTER) and the scatter plots were acquired for some selected cancers.

PLOD3 was put in the Sangerbox tool (http://sangerbox.com/Tool, accessed on 30 May 2021) to explore the potent relation of the PLOD3 expression to TMB or MSI across different cancers from TCGA database. Furthermore, the relationship between PLOD3 expression and immune cells was also explored.

## Data Availability

The data provided in this study can be obtained in the method section of this manuscript. The results shown here are, in part, based upon data generated by TCGA Research Network (https://www.cancer.gov/tcga), GTEx database (https://commonfund.nih.gov/gtex) and HPA database (https://www.proteinatlas.org/humanproteome/pathology).

## Supplementary Materials

The following are available online at https://www.mdpi.com/article/10.3390/ijms22189903/s1.
