# Supplementary material for: A Human Pan-Cancer System Analysis of Procollagen-Lysine, 2-Oxoglutarate 5-Dioxygenase 3 (PLOD3)
Source: Int J Mol Sci. 2021 Sep 14;22(18):9903. doi: 10.3390/ijms22189903 (PMC8467482; doi:10.3390/ijms22189903)
Supplement: Supplementary file 1 [file ijms-22-09903-s001.zip › ijms-1339281-supplementary.pdf]

International Journal of
Molecular Sciences

MDPI

Supplementary Material

A Human Pan-cancer System Analysis of Procollagen-lysine, 2-oxoglutarate 5-dioxygenase 3 (PLOD3)

Siming Gong [1], Yingjuan Duan [2], Changwu Wu [1,*], Georg Osterhoff [3], Nikolas Schopow [1,3,†] and Sonja Kallendrusch [1,†]

[1] Institute of Anatomy, University of Leipzig, Liebigstraße 13, 04103 Leipzig, Germany; siminggong23@gmail.com (S.G.); nikolas.schopow@medizin.uni-leipzig.de (N.S.) sonja.kallendrusch@medizin.uni-leipzig.de (S.K.);
[2] Faculty of Chemistry and Mineralogy, University of Leipzig, 04103 Leipzig, Germany; yingjuan0103@gmail.com
[3] Sarcoma Center, Department of Orthopedics, Trauma and Plastic Surgery, University Hospital Leipzig, 04103 Leipzig, Germany; georg.osterhoff@medizin.uni-leipzig.de
* Correspondence: wuchangwu93@gmail.com
† These authors contributed equally to this work and share the last authorship.

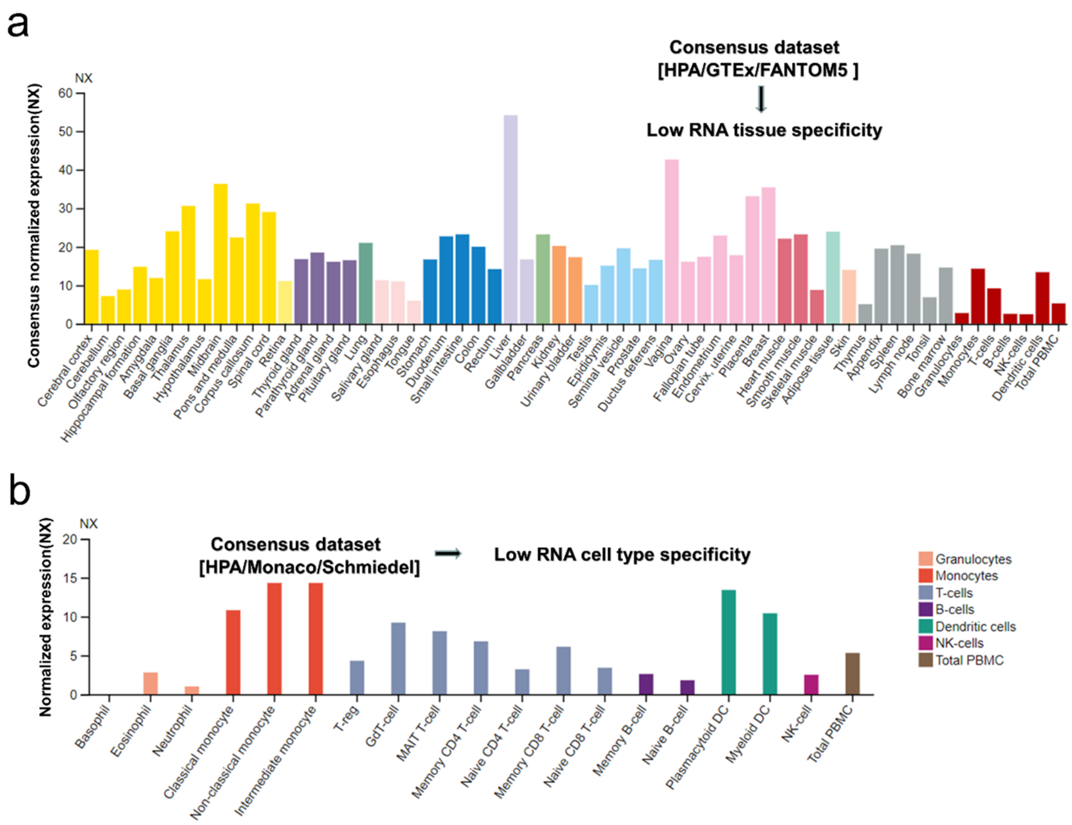

Figure S1. Expression level of PLOD3 in different normal tissues and blood cells. (a) PLOD3 gene in different normal tissues using the consensus datasets of HPA, GTEx and FANTOM5. (**b**) PLOD3 gene in different normal blood cells using the consensus datasets of HPA, Monaco and Schmiedel.

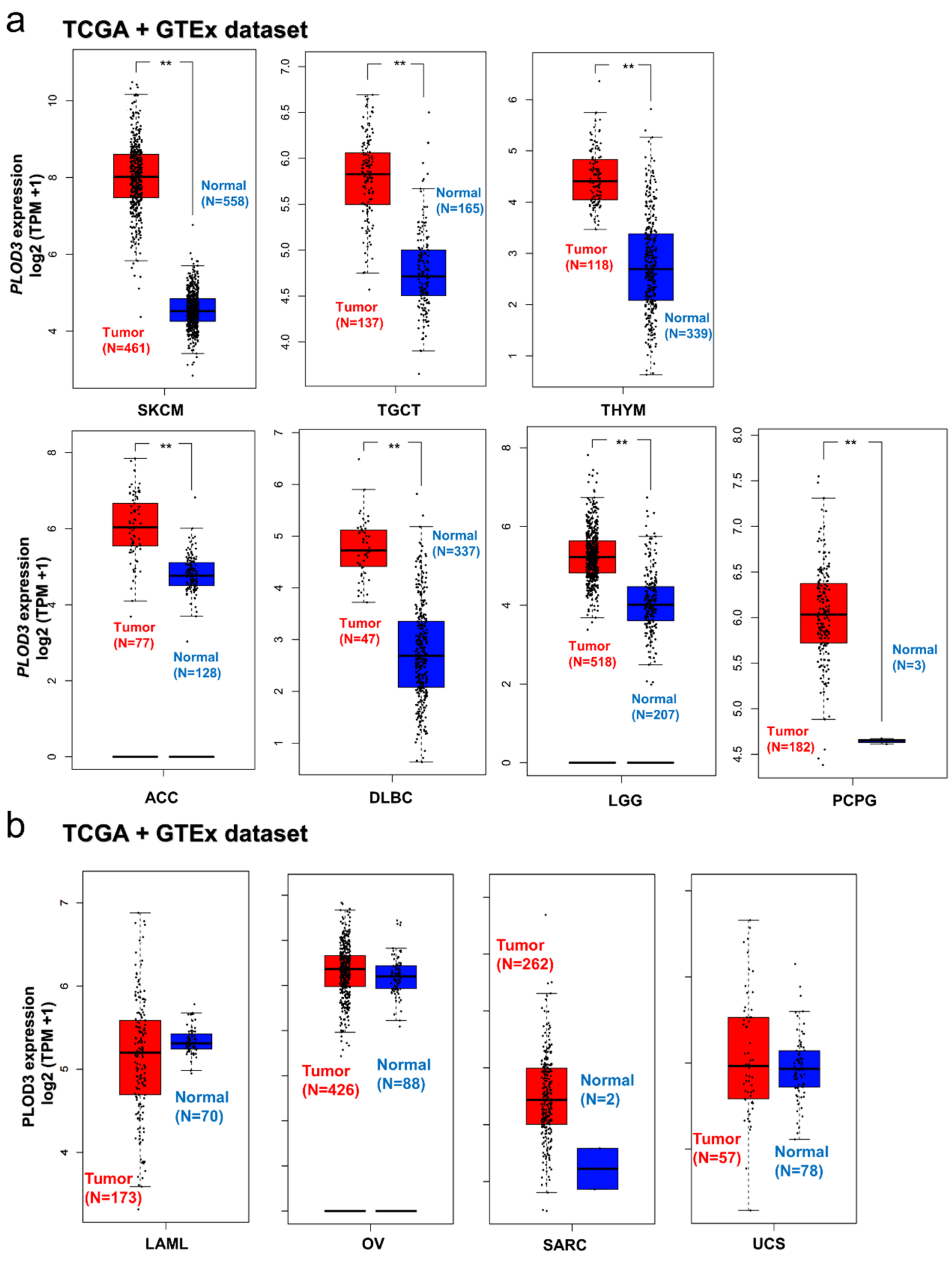

Figure S2. PLOD3 expression in different cancers (**a**) The normal tissues from the GTEx database were included as controls for ACC, DLBC, LGG, PCPG, SKCM, TGCT and THYM in the TCGA project. The box plot data were supplied. ** p <0.01. (**b**) The normal tissues from the GTEx database compare with tumor tissue from TCGA for LAML, OV, SARC and UCS.

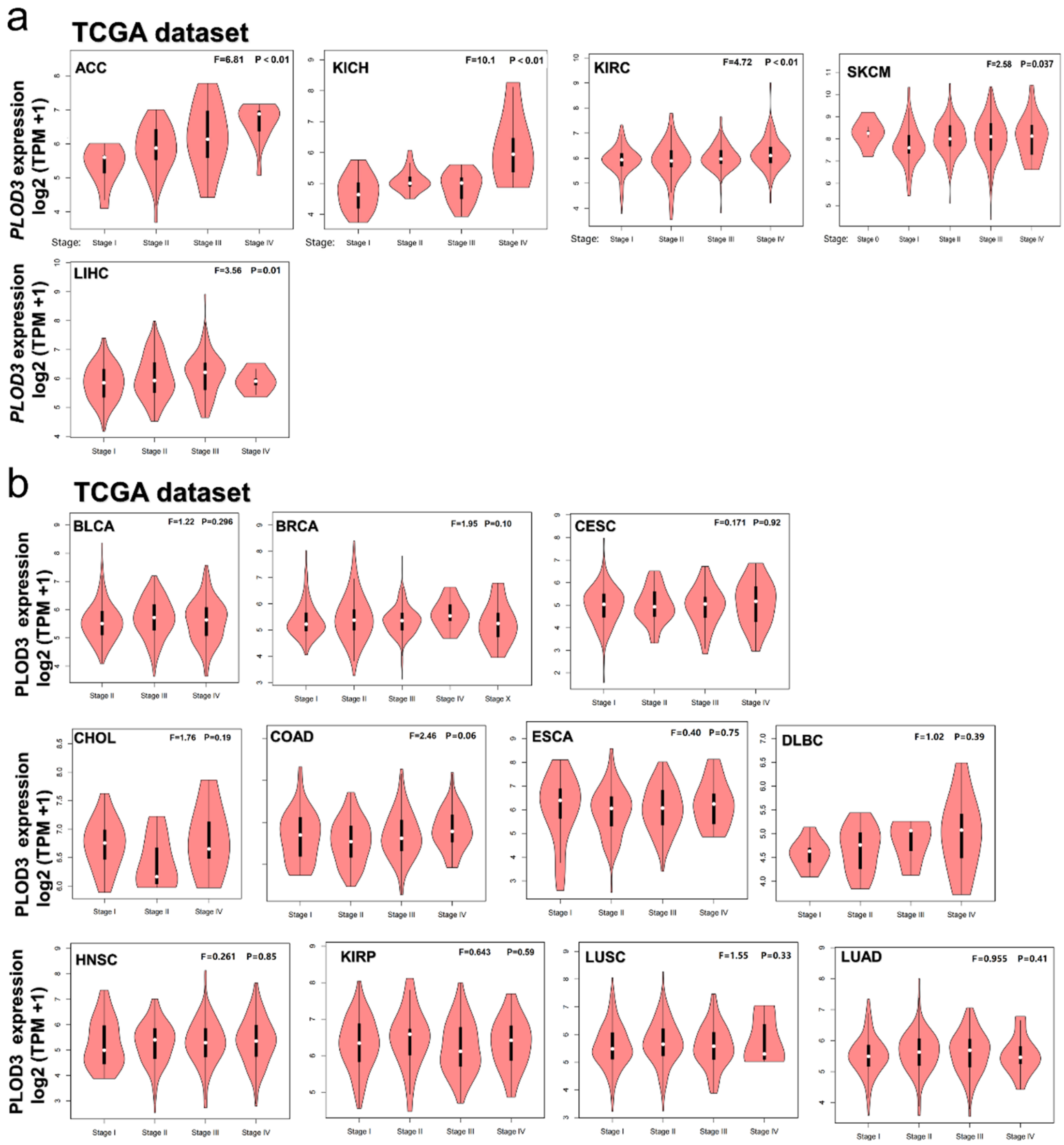

Figure S3. PLOD3 expression in different pathological stages. The correlation between PLOD3 expression and different pathological stages. (a) The expression of PLOD3 were analyzed by the main pathological stages of ACC, KICH, KIRC, SKCM and LIHC. (b) Expression levels of the PLOD3 gene by different pathological stages of BLCA, BRCA, CESC, CHOL, COAD, ESCA, DLBC, HNSC, KIRP, LUSC and LUAD.

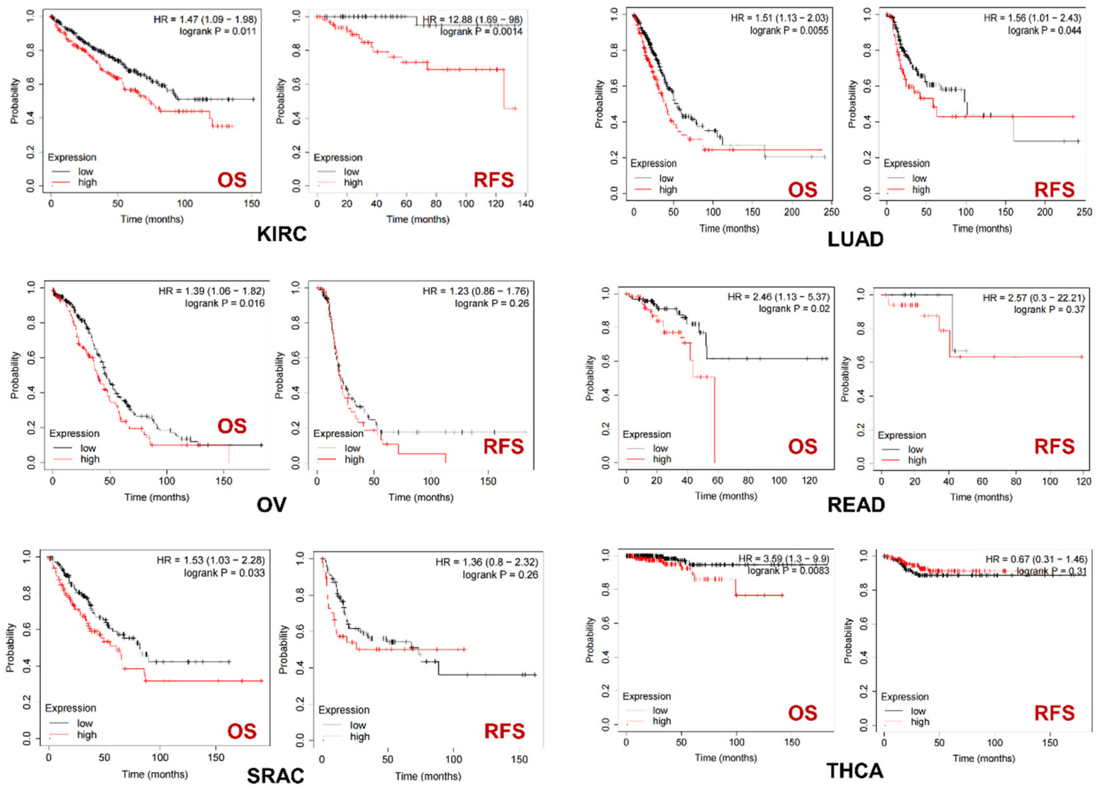

Figure S4. Correlation between PLOD3 gene expression and prognosis of cancers was analyzed by Kaplan Meier plotter. OS–overall survival, RFS- relapse free survival.

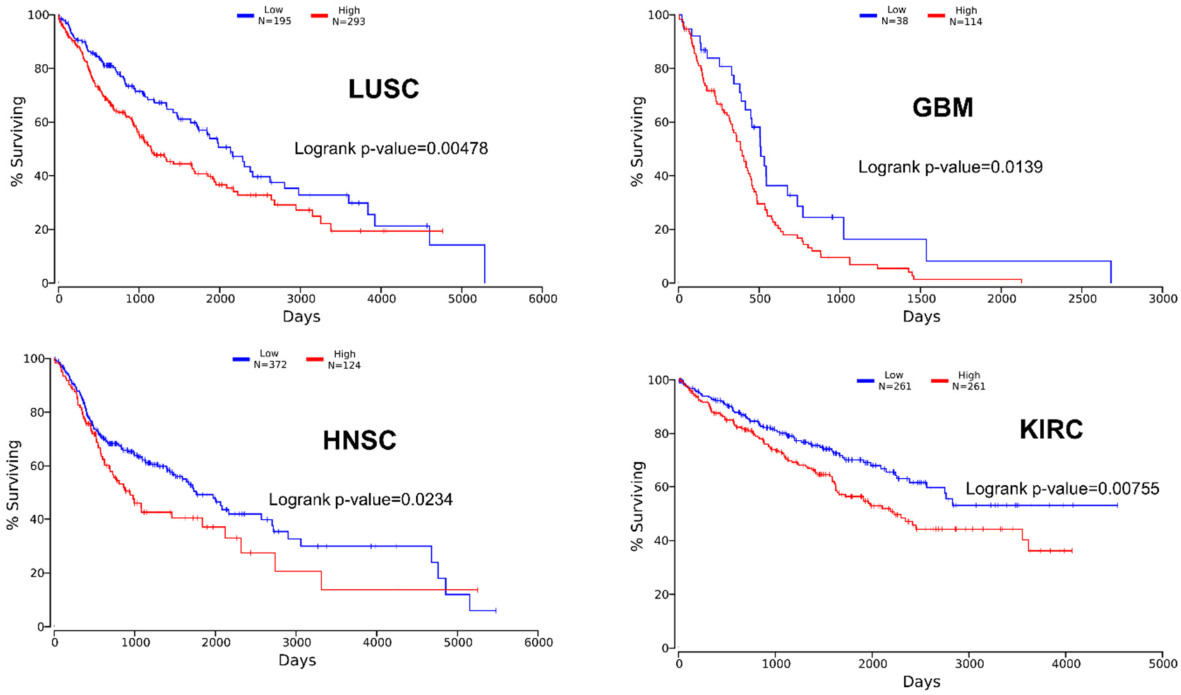

Figure S5. Oncolnc tool was used to analyzed the correlation between PLOD3 expression and prognosis (OS).

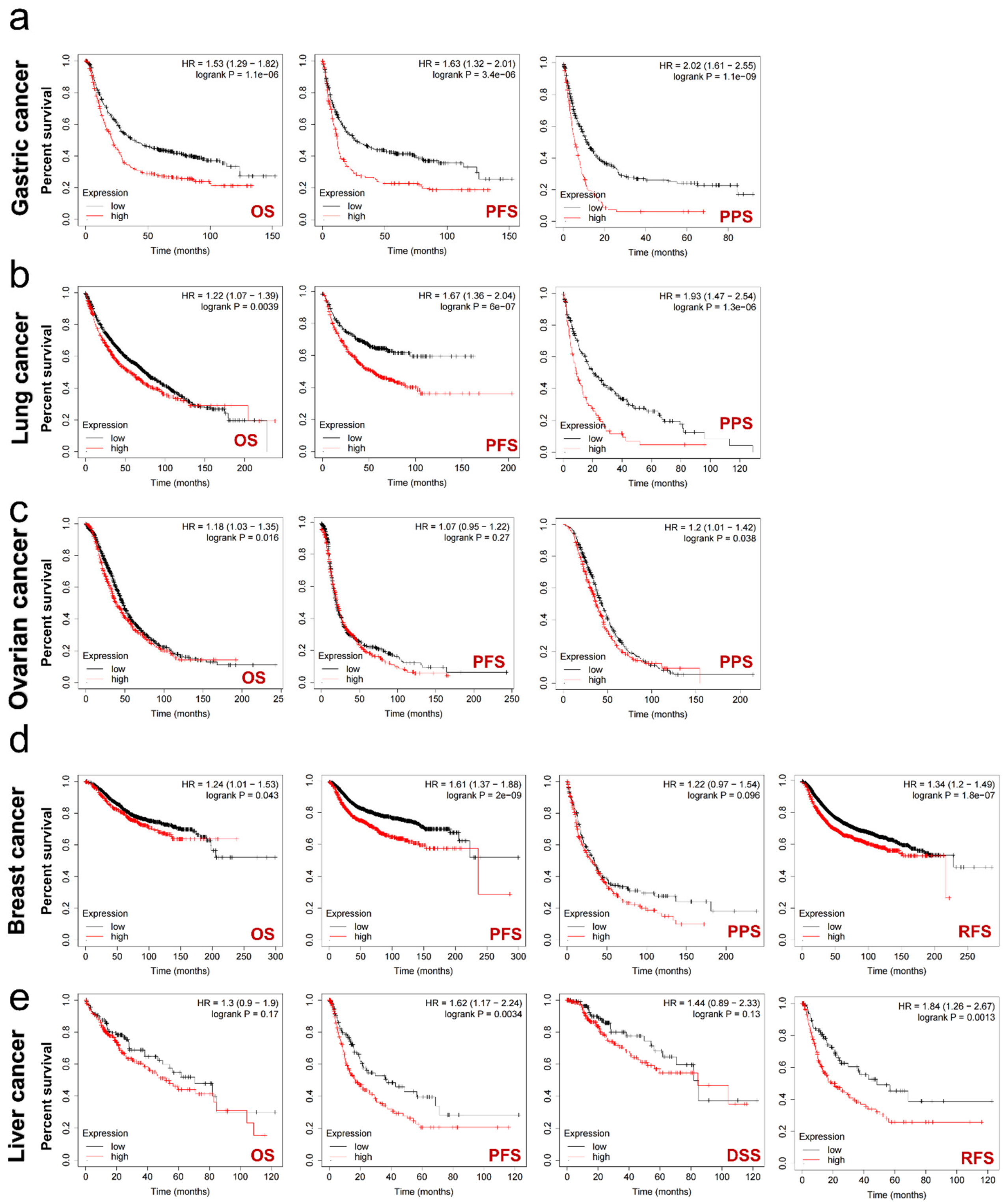

Figure S6. Correlation between PLOD3 gene expression and prognosis of cancers. Series of survival analyses in (**a**) Gastric cancer, (**b**) lung cancer, (**c**) ovarian cancer, (**d**) breast cancer and (**e**) liver cancer. OS–overall survival, RFS—relapse free survival, PFS—progression free survival, PPS- post progression survival, and DSS—disease specific survival.

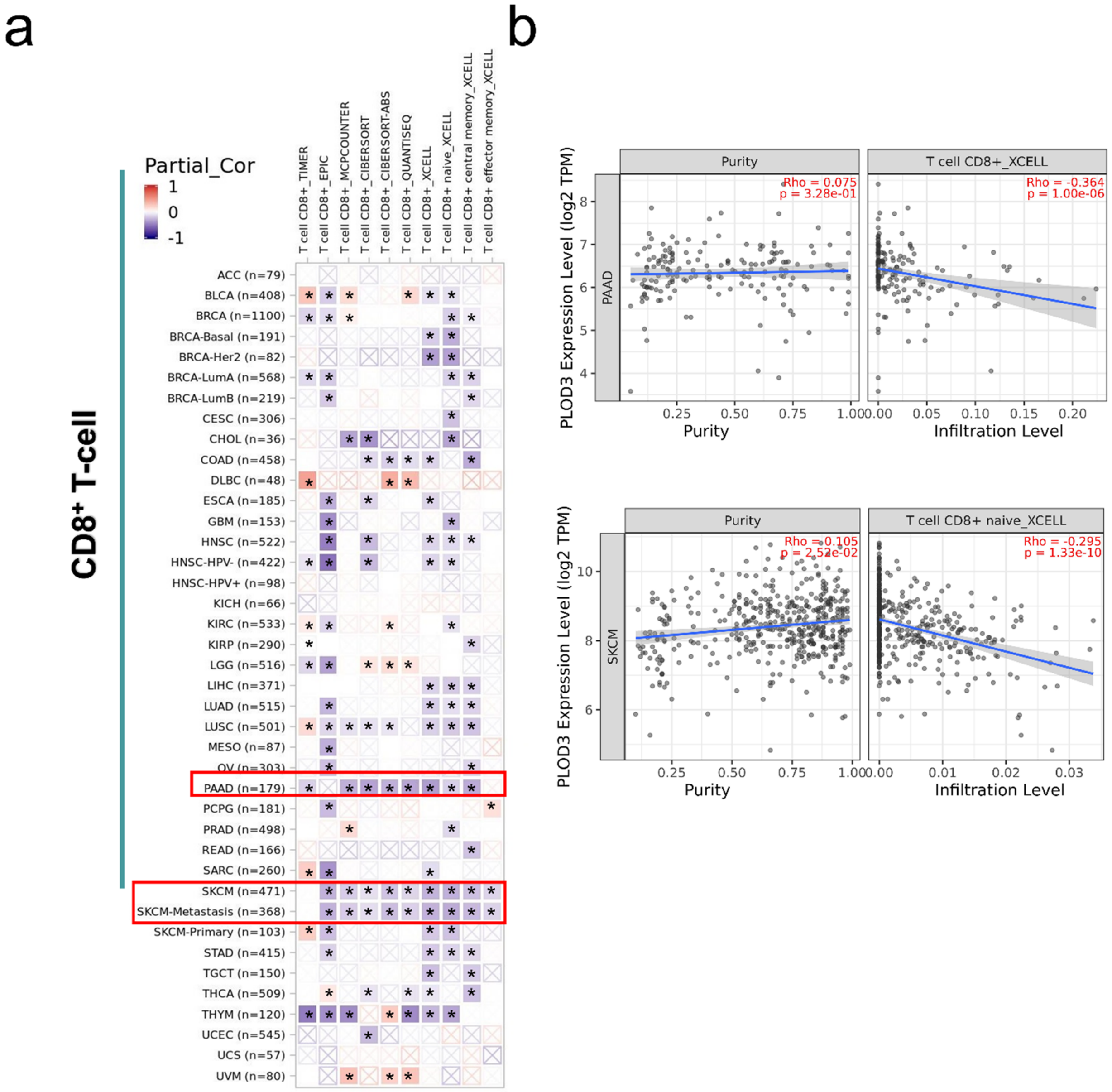

Figure S7. Correlation analysis between *PLOD3* expression and immune infiltration of CD8+ T-cells. We used different algorithm to obtain the potential correlation between the expression level of *PLOD3* gene and the infiltration level of CD8+ T-cells across types of cancer in TCGA. * $p < 0.05$.

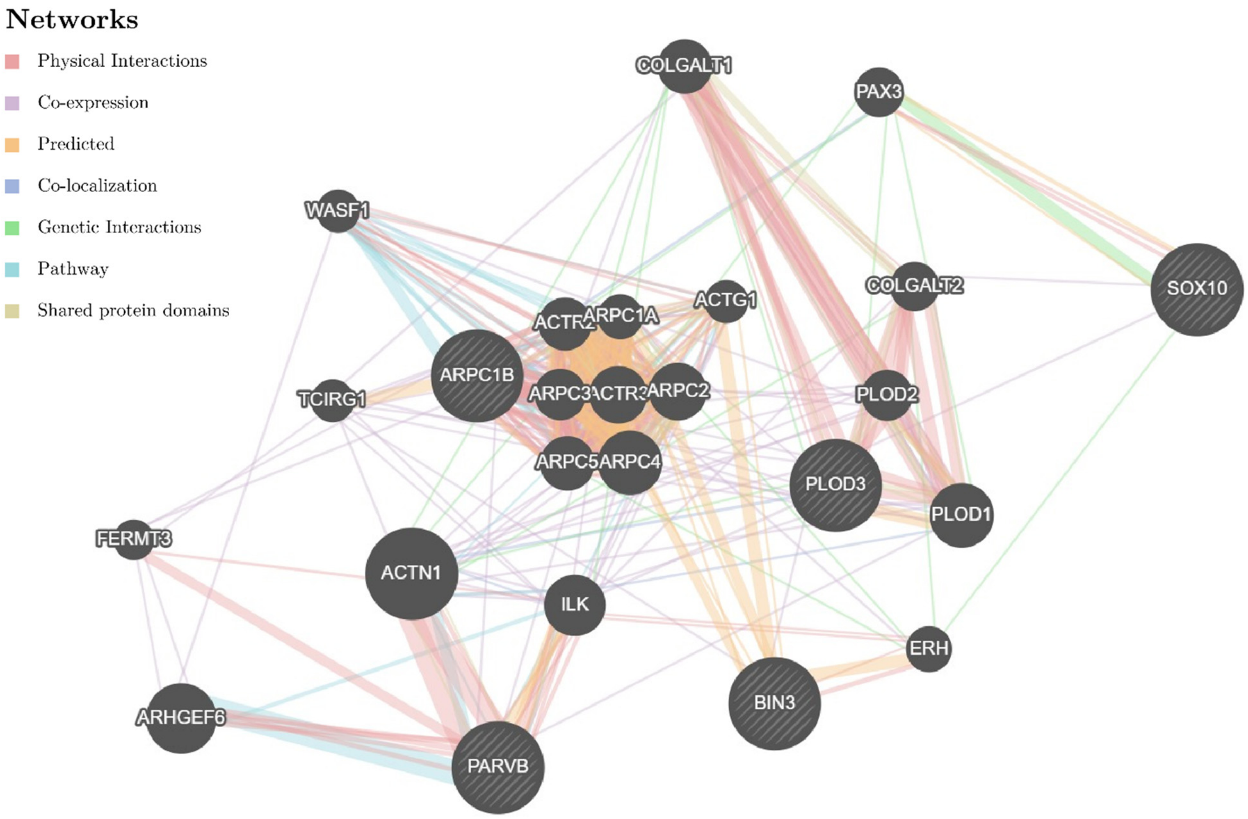

Figure S8. Network of PLOD3 was analyzed by GeneMANIA.
